# Structure-based engineering of the midnolin-proteasome pathway for targeted protein degradation

**DOI:** 10.1093/procel/pwaf069

**Published:** 2025-08-20

**Authors:** Hongyang Wang, Ying Zheng, Tiantian Wang, Xue Zhang, Peipei Wang, Chuancun Wei, Hongyue Li, Quan Wang, Lu Zhang, Xisong Ke, Wenqing Xu

**Affiliations:** School of Life Science and Technology, ShanghaiTech University, Shanghai 201210, China; School of Life Science and Technology, ShanghaiTech University, Shanghai 201210, China; Center for Chemical Biology, Institute of Interdisciplinary Integrative Medicine Research, Shanghai University of Traditional Chinese Medicine, Shanghai 201203, China; Center for Chemical Biology, Institute of Interdisciplinary Integrative Medicine Research, Shanghai University of Traditional Chinese Medicine, Shanghai 201203, China; Shanghai Institute for Advanced Immunochemical Studies, ShanghaiTech University, Shanghai 201210, China; School of Life Science and Technology, ShanghaiTech University, Shanghai 201210, China; Shanghai Institute for Advanced Immunochemical Studies, ShanghaiTech University, Shanghai 201210, China; School of Life Science and Technology, ShanghaiTech University, Shanghai 201210, China; School of Life Science and Technology, ShanghaiTech University, Shanghai 201210, China; Shanghai Institute for Advanced Immunochemical Studies, ShanghaiTech University, Shanghai 201210, China; Shanghai Clinical Research and Trial Center, Shanghai 201203, China; School of Life Science and Technology, ShanghaiTech University, Shanghai 201210, China; Shanghai Institute for Advanced Immunochemical Studies, ShanghaiTech University, Shanghai 201210, China; Shanghai Clinical Research and Trial Center, Shanghai 201203, China; Center for Chemical Biology, Institute of Interdisciplinary Integrative Medicine Research, Shanghai University of Traditional Chinese Medicine, Shanghai 201203, China; School of Life Science and Technology, ShanghaiTech University, Shanghai 201210, China

## Dear Editor,

Targeted protein degradation (TPD) through proteasomal pathways has become a powerful tool for biomedical development (Tsai et al., 2024). PROTACs and molecular glues use E3 ligases to polyubiquitinate substrates for degradation (Békés et al., 2022). However, ubiquitination-dependent approaches face several challenges in their therapeutic use (Tsai et al., 2024). Firstly, only a few human E3 ligases have been successfully utilized for TPD, and their expression varies across cells (Békés et al., 2022; Khan et al., 2019; Zhao et al., 2022). Secondly, numerous deubiquitinating enzymes (DUBs) (Snyder and Silva, 2021) in targeted cells may complicate the degradation efficiency and promote drug resistance. Thirdly, it is difficult to achieve subcellular-specific degradation, potentially increasing off-target toxicity. As the proteasome exists ubiquitously in all known cell types, ubiquitination-independent proteasomal degradation may be a solution to these challenges.

**Figure 1.**
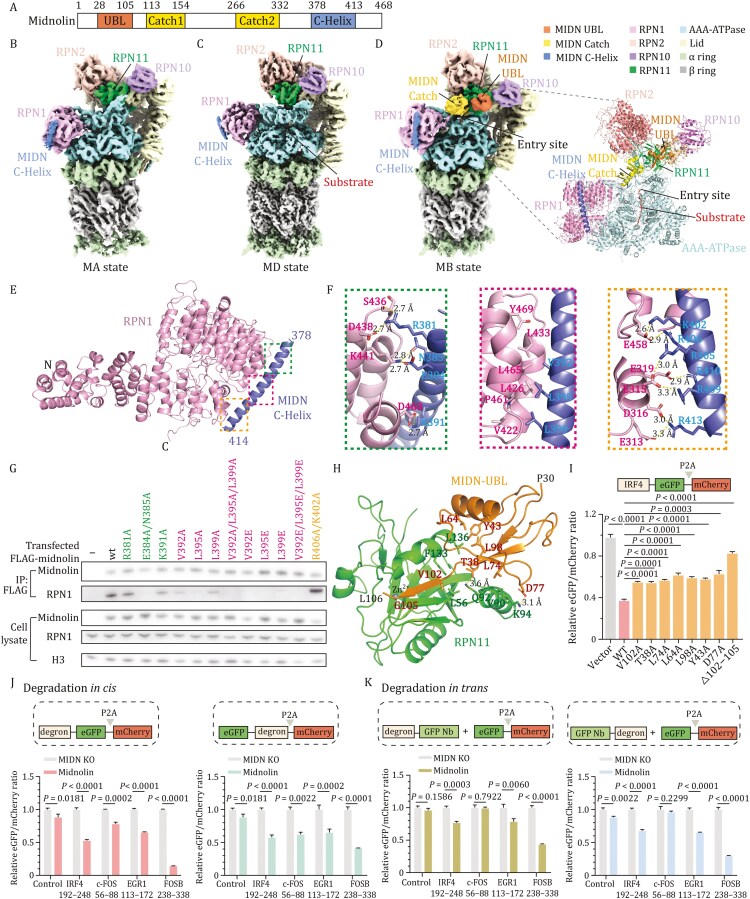
Structural and mechanistic analysis of protein-degradation mediated by the midnolin-proteasome system. (A) Domain organization of the human midnolin. (B and C) Cryo-EM density maps of the midnolin-proteasome complex in the MA and MD states, respectively. (D) Cryo-EM density map (left) of midnolin-proteasome complex in the MB state and cartoon structure (right) of midnolin and partial RP subunits. While cryo-EM densities for MIDN-UBL and C-Helix are clearly enough for model building, the density for MIDN-Catch in the MB state is visible but not clear enough to allow precise model building. Thus, an AlphaFold3 model for the Catch domain is roughly fitted into the density. (E) Structural illustration of the MIDN C-Helix–RPN1 interaction. (F) Close-up views of specific interactions between MIDN C-Helix and RPN1. RPN1 and midnolin are colored in pink and slate, respectively. (G) Specific residues within the MIDN C-Helix are required for midnolin’s interaction with RPN1, as shown by Co-IP assays. The mutants are color-coded by corresponding regions in the C-Helix, same as in (E). (H) Structural illustration of the midnolin-UBL–RPN11 interaction and key interface residues. (I) Midnolin residues mediating the UBL–RPN11 interaction are critical for promoting substrate degradation. The indicated wild-type full-length midnolin or its mutants were transfected into MIDN KO HEK293T cells with an IRF4-eGFP-P2A-mCherry plasmid. Protein stability was measured as the eGFP/mCherry ratio. The empty vector serves as a control. Error bars indicate standard deviations; unpaired two-tailed *t* tests were used for statistical analyses. (J) The FOSB degron has the best degradation capacity *in cis* among four different midnolin degrons. IRF4, c-FOS, EGR1, and FOSB peptide, which encompass the predicted β strand bound to the midnolin Catch domain, were either directly fused to the N-terminal (left) or C-terminal (right) side of eGFP to assess degradation efficiencies *in cis*. eGFP fusions were transfected into MIDN KO HEK293T cells with/without midnolin plasmids, and eGFP/mCherry ratios were measured as indicators of corresponding protein stabilities. Error bars indicate standard deviations; unpaired two-tailed t tests were used for statistical analyses. (K) The FOSB degron has the best degradation capacity *in trans* among four different midnolin degrons. Four midnolin degrons were either fused to the N-terminal (left) or C-terminal (right) side of a nanobody against eGFP for degradation *in trans*. EGFP-Nb fusions were co-transfected with the eGFP-P2A-mCherry plasmid into MIDN KO HEK293T cells with/without midnolin plasmids. Fluorescence was measured with flow cytometry and normalized to corresponding midnolin non-transfected pairs. Error bars indicate standard deviations; unpaired two-tailed *t* tests were used for statistical analyses.

Midnolin was discovered to promote degradation of non-ubiquitinated proteins in a ubiquitin-independent manner, by directly recruiting substrates to proteasomes (Gu et al., 2023). As a critical nuclear regulator of protein homeostasis, midnolin mediates precise and rapid degradation of substrates in the nucleus, such as immediate early gene-encoded proteins and other cell type-specific transcriptional regulators such as IRF4, responding to dynamic changes in intracellular stress and metabolic signals (Gu et al., 2023). Thus, the midnolin-proteasome pathway provides a template for developing novel TPD strategies through direct 26S proteasome recruitment. Midnolin features an N-terminal ubiquitin-like (UBL) domain important for substrate degradation, a Catch domain responsible for substrate recruitment, and a C-terminal α-helix (C-Helix), which mediates interactions with the proteasome (Gu et al., 2023) (Figs. 1A and S1). How midnolin interacts with the proteasome and promotes substrate degradation remains enigmatic, hindering its potential application.

To understand how midnolin interacts with 26S proteasome to mediate substrate degradation, we determined the cryo-EM structures of midnolin in complex with 26S proteasome (Figs. 1, S2 and S3). Unsupervised 3D classification identified three distinct states, designated MA, MB, and MD, with nominal resolutions of 4.32 Å, 3.76 Å, and 3.31 Å, respectively (Figs. 1B–D and S4; Table S1). All three states show clear density for midnolin C-Helix, but only the MB state shows clear density for the UBL domain of midnolin and weak density for the Catch domain of midnolin.

Both the AAA-ATPase channel in the regulatory particle (RP) and the 20S core particle gate are closed in the MA state. For the MB state, its lid is rotated ~15˚ outwards away from the axis of ATPase relative to the MA state, which enlarges the entrance to the ATPase channel (Fig. S5). The RP of MD state undergoes a large-scale conformational change, which results in the alignment of the translocation channel within the RP and the open core particle gate to facilitate substrate degradation. We found that MA, MB, and MD states resemble the canonical ubiquitinated substrate-engaged states E_A1_, E_B_, and E_D2_ (Fig. S5A), key functional conformations for ubiquitin recognition, deubiquitylation, and processive degradation, respectively (Dong et al., 2019). Notably, in the MB state, MIDN-UBL binds to RPN11 and its adjacent Catch domain located above the AAA-ATPase entry site to facilitate substrate translocation and unfolding (Fig. 1D).

Our cryo-EM structure clearly shows that the midnolin C-Helix binds to the T2 site of RPN1 subunit within 26S proteasome (Figs. 1E, S6A and S6B) through three sets of contacts, with a hydrophobic core buttressed by hydrophilic interactions on both sides, burying an interface area of 1,133.5 Å^2^ (Fig. 1F). Using a biolayer interferometry (BLI) assay, we measured the affinity between full-length RPN1 and GST-tagged midnolin C-Helix (375–413), revealing a tight interaction with a *K*_D_ of ~88.5 nmol/L (Fig. S6C).

To identify interface hotspots, we generated 10 mutants on the midnolin C-Helix residues contacting RPN1 (Figs. 1G and S6E). Single or triple mutants of V^392^/L^395^/L^399^ strongly reduced or completely abolished the binding of midnolin to 26S proteasome, underscoring the importance of this hydrophobic core. N-terminal mutant (E384A/N385A) strongly reduced the binding of midnolin to 26S proteasome (Fig. 1G). In contrast, C-terminal mutants (R406A/K402A, R410A/R413A, and R405A/R409A) did not significantly affect binding (Figs. 1G and S6E), consistent with the primary function of the ^402^KRLRRKARR^410^ segment as a nuclear localization signal (Gu et al., 2023; Tsukahara et al., 2000). Furthermore, consistent with our Co-IP results, mutants that disrupt the interaction of C-Helix with RPN1 significantly reduce the ability of midnolin to promote the degradation of IRF4 degron fused eGFP (Fig. S6F).

In the MB state, our cryo-EM structure clearly shows that the UBL domain of midnolin binds to the RPN11 subunit (Figs. 1D, S7A and S7B), a deubiquitinase subunit within RP with a known ubiquitin-binding site (Fig. S7C and S7D; Worden et al., 2017). We found that the highly conserved C-terminal tail of UBL (residue 102–105) forms an antiparallel three-stranded β sheet with the RPN11 Ins-1 loop. And UBL Y43, L64, and L98 form a hydrophobic pocket with RPN11 L136. UBL L74 forms hydrophobic interactions with RPN11 V90. In addition, UBL T38 and D77 form hydrogen bonds with RPN11 Q92 and K94, respectively. In support of this MIDN-UBL/RPN11 interaction model, deletion of UBL_102–105_ and mutagenesis of MIDN interface residues impair the ability of midnolin to promote the degradation of IRF4 degron fused eGFP (Fig. 1I). Overall, our structural studies of the proteasome-midnolin complex revealed that midnolin’s UBL domain and C-Helix interact with the proteasome RPN11 and RPN1 subunits, respectively, aligning its substrate-binding Catch domain above the proteasome ATPase motor, thus facilitating substrate degradation.

Next, we characterized degrons of known midnolin substrates (Fig. S8) and tested if midnolin can mediate the degradation of proteins indirectly recruited by midnolin. Our results showed that the FOSB degron is the most effective one among the tested degrons, and that midnolin can mediate the degradation of substrates directly (*in cis*) or indirectly (*in trans*) by the FOSB degron (Fig. 1J and 1K). Thus, our work suggested that the “two-arm” midnolin system is highly effective and can potentially be engineered for the degradation of other proteins.

**Figure 2.**
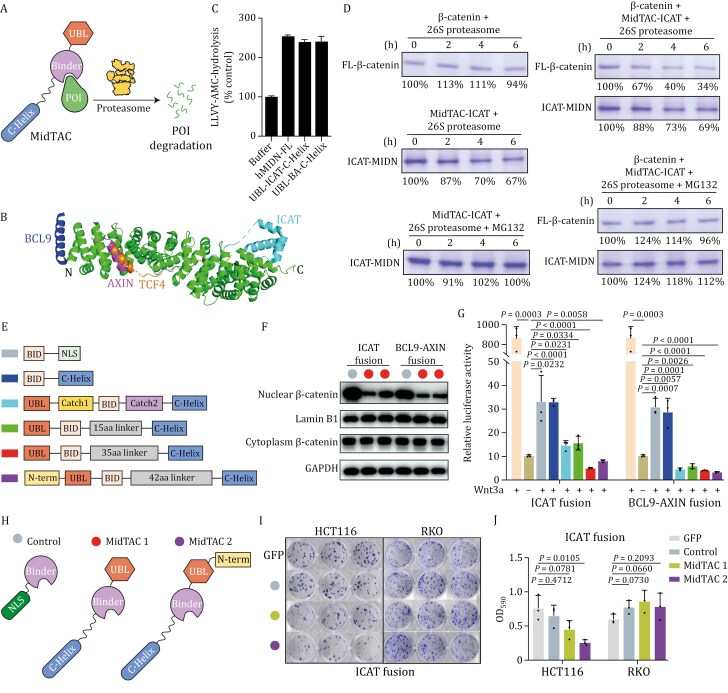
Engineered midnolin targeting chimeras (MidTAC) can efficiently promote targeted protein degradation *in vitro* and in live cells. (A) Schematic representation of engineered midnolin-mediated degradation. POI: protein of interest. (B) Structure superposition of β-catenin with its binders: β-catenin/ICAT complex (Graham et al., 2002) (PDB:1LUJ), β-catenin/AXIN complex (Xing et al., 2003) (PDB:1QZ7), and β-catenin/BCL9/TCF4 complex (Sampietro et al., 2006) (PDB:2GL7). (C) Engineered midnolin has comparable capacity to activate 26S proteasome as wt midnolin, as shown by *in vitro* peptidase activity of purified 26S proteasomes (1 nmol/L) and measured by AMC fluorescence after hydrolysis of LLVY-AMC (10 μmol/L). Buffer was used as a control. (D) Purified engineered midnolin mediated degradation of purified β-catenin in the presence of purified human 26S proteasomes *in vitro*, and MG132 inhibits this degradation. PAGE gel stained by Coomassie Brilliant Blue. (E) Schematic representation of various engineered midnolins. (F) MidTAC-mediated degradation of nuclear β-catenin in HEK293T cells, but not cytoplasmic β-catenin, as shown by Western blot. Lamin B1 and GAPDH were used as loading controls. (G) MidTAC-mediated inhibition of Wnt/β-catenin signaling activity. Various MidTACs were transfected into HEK293T cells, and the Wnt/β-catenin signaling activity was measured by TOPflash assays. Error bars indicate standard deviations; unpaired two-tailed *t* tests were used for statistical analyses. (H) Schematic representation of control and MidTACs used in (I and J). MidTAC 1 and MidTAC 2 represent the constructs shown as red and purple in (E), respectively. (I) Representative images of clone formation assays for HCT116 and RKO cells after transfection with the indicated lentivirus. (J) Quantification of clone formation ability of HCT116 and RKO cells after transfection with the indicated lentivirus, as shown in (I). Error bars indicate standard deviations; unpaired two-tailed *t* tests were used for statistical analyses.

Therefore, we hypothesized that an engineered midnolin, when its Catch domain is replaced with a binding domain/motif of a specific target protein, may function and degrade the specific target protein in the nucleus, named as engineered midnolin targeting chimeras (MidTAC; Fig. 2A). To test this hypothesis, we specifically tested the degradation of nuclear β-catenin, which is a multifunctional protein. While cadherin-associated β-catenin near the membrane connects adherens junctions to the cytoskeleton and serves as a tumor suppressor (Grimson et al., 2000), nuclear β-catenin activates Wnt-target genes and promotes tumorigenesis (Nusse and Clevers, 2017). As such, for cancer treatment and mechanistic analysis of Wnt signaling, approaches to specifically deplete nuclear β-catenin are highly desirable. To substitute midnolin Catch domain, we reconstituted two previously reported β-catenin interacting domains (BID; Graham et al., 2002; Sampietro et al., 2006; Xing et al., 2003), namely ICAT^1–81^ and BCL9^347–392^-AXIN^436–498^ fusion (BA; Fig. 2B). Both purified MidTACs can stimulate 26S peptidase activity with a level similar to that of wild-type midnolin (Fig. 2C).

To directly test whether the MidTAC for β-catenin has the ability to specifically degrade nuclear β-catenin, we reconstituted an *in vitro* degradation system, using MidTAC and β-catenin purified from *E. coli*, and human 26S proteasome purified from HEK293T cells (Figs. 2D and S9). Addition of β-catenin alone did not result in its degradation, while addition of MidTAC-ICAT alone resulted in its partial degradation, and this degradation was blocked by MG132, a proteasome inhibitor (Fig. 2D). Importantly, when both MidTAC-ICAT and β-catenin were added to the reaction, β-catenin was largely degraded and MidTAC-ICAT was partially degraded along with β-catenin, and MG132 inhibited β-catenin degradation (Fig. 2D). Thus we showed that MidTAC-ICAT can promote proteasomal β-catenin degradation in reconstituted systems.

To interrogate whether MidTAC can promote nuclear β-catenin degradation in live cells, we generated various MidTAC constructs (Fig. 2E). We designed a short linker of 15 amino acids between MidTAC-UBL and BID to position β-catenin close to the ATPase motor. And we also designed different lengths of linkers between BID and C-Helix to ensure sufficient flexibility for MidTAC to function effectively. We fractionated HEK293T cells overexpressing MidTAC. The results showed that both MidTACs significantly reduced nuclear β-catenin levels, but had little effect on cytoplasmic β-catenin levels (Fig. 2F), demonstrating the efficiency and specificity of MidTACs.

Using Topflash assays, we tested MidTACs’ ability to inhibit Wnt signaling. BIDs alone inhibited Wnt signaling, as they can interfere with β-catenin and transcription factor interactions (Fig. 2G). BID fused to C-Helix has a similar inhibitory effect on Wnt signaling as BID itself, probably because fusing C-Helix alone is unable to degrade β-catenin efficiently. However, “two-arm” MidTACs enhanced the inhibitory effect on Wnt signaling and reduced the signal even below the non-activated state (Fig. 2G), likely combining the effect of transcriptional complex assembly inhibition and nuclear β-catenin degradation. Next, we further explored their β-catenin-inhibition abilities in colorectal cancer cell lines (Fig. 2H). MidTAC significantly suppressed proliferation of the HCT116 cell line (which has abnormally upregulated Wnt/β-catenin activity), but not the RKO cell line (which has a low basal level of β-catenin; Fig. 2I and 2J), demonstrating β-catenin-dependent activity.

In conclusion, our structural and functional data reveal how midnolin mediates ubiquitination-independent substrate degradation (Fig. S10A). Based on structural and mechanistic understanding of midnolin-proteasome pathway, we developed MidTAC, a ubiquitination-independent TPD approach (Fig. S10B). As proof of concept, we targeted β-catenin for proteasomal degradation by generating a heterobifunctional MidTAC composed of the N-terminal UBL, a β-catenin binder, and the C-Helix. We show that MidTAC effectively degraded β-catenin *in vitro*. Notably, MidTACs specifically degrade nuclear β-catenin, but not cytoplasmic β-catenin in tested cell lines. Thus, this approach provides therapeutic potential for traditionally “undruggable” proteins, such as β-catenin.

MidTAC presents significant advantages over conventional TPD approaches. Firstly, as a ubiquitination-independent system, it can degrade proteins that do not have lysine residues, significantly expanding the range of targets. Secondly, since the 26S proteasome is abundant and ubiquitous, MidTAC can degrade target proteins across all cell/tissue types, and may specifically degrade target proteins located in a desired subcellular compartment (with a localization signal). Thirdly, as optimized protein binders can be more specific than a small molecule binder, MidTAC can achieve remarkable specificity to minimize off-target effects.

## Footnotes

We thank Qianqian Sun for technical support in cryo-EM data collection at the Bio-Electron Microscopy Facility of ShanghaiTech University.

This work was funded by the Natural Science Foundation of Shanghai (24ZR1451800) to H.W., Chinese Academy of Sciences Pilot Strategic Science and Technology Projects B grant (XDB37030302), and a start-up fund from the ShanghaiTech University (2018F0202-000-11) to W.X. We thank the Shanghai Frontiers Science Center for Biomacromolecules and Precision Medicine, Shanghaitech University.

W.X., H.W., Y.Z., T.W., and X.K. have a patent application related to this work. X.Z., P.W., C.W., H.L., Q.W., and L.Z declare that they have no conflict of interest. The authors declare their agreement to publish.

Cryo-EM density maps of midnolin-proteasome complexes determined in this study have been deposited in the Electron Microscopy Data Bank under accession numbers EMD-64103 (MA), EMD-64133 (MB), EMD-65264 (MB with better Catch domain density), and EMD-63592 (MD). The corresponding atomic structure coordinates have been deposited in the Protein Data Bank under accession codes 9UF8 (MA), 9UG9 (MB), and 9M2W (MD).

W.X. and H.W. conceived the project and designed the experiments. H.W. purified the protein complexes, prepared the cryo-EM samples, and collected and processed cryo-EM data. H.W., P.W., and Q.W. refined the density maps. H.W., C.W., and L.Z. built and refined the atomic models. H.W., Y.Z., and H.L. conducted the biochemical experiments and protein degradation assays. X.Z. prepared the RPN11 stable cell line and T.W. performed the colony formation assay under the supervision of X.K. W.X. supervised this study. H.W. and W.X. analyzed the data and wrote the manuscript, and all authors revised the manuscript.

## Supplementary Material

pwaf069_Supplementary_Materials_1

## References

[CIT0001] Békés M, Langley DR, Crews CM. PROTAC targeted protein degraders: the past is prologue. Nat Rev Drug Discov 2022;21:181–200. 35042991 10.1038/s41573-021-00371-6 PMC8765495

[CIT0002] Dong Y, Zhang S, Wu Z et al Cryo-EM structures and dynamics of substrate-engaged human 26S proteasome. Nature 2019;565:49–55. 30479383 10.1038/s41586-018-0736-4 PMC6370054

[CIT0003] Graham TA, Clements WK, Kimelman D et al The crystal structure of the β-catenin/ICAT complex reveals the inhibitory mechanism of ICAT. Mol Cell 2002;10:563–571. 12408824 10.1016/s1097-2765(02)00637-8

[CIT0004] Grimson MJ, Coates JC, Reynolds JP et al Adherens junctions and β-catenin-mediated cell signalling in a non-metazoan organism. Nature 2000;408:727–731. 11130075 10.1038/35047099

[CIT0005] Gu X, Nardone C, Kamitaki N et al The midnolin-proteasome pathway catches proteins for ubiquitination-independent degradation. Science 2023;381:eadh5021. 37616343 10.1126/science.adh5021 PMC10617673

[CIT0006] Khan S, Zhang X, Lv D et al A selective BCL-XL PROTAC degrader achieves safe and potent antitumor activity. Nat Med 2019;25:1938–1947. 31792461 10.1038/s41591-019-0668-z PMC6898785

[CIT0007] Nusse R, Clevers H. Wnt/β-catenin signaling, disease, and emerging therapeutic modalities. Cell 2017;169:985–999. 28575679 10.1016/j.cell.2017.05.016

[CIT0008] Sampietro J, Dahlberg CL, Cho US et al Crystal structure of a β-catenin/BCL9/Tcf4 complex. Mol Cell 2006;24:293–300. 17052462 10.1016/j.molcel.2006.09.001

[CIT0009] Snyder NA, Silva GM. Deubiquitinating enzymes (DUBs): Regulation, homeostasis, and oxidative stress response. J Biol Chem 2021;297:101077. 34391779 10.1016/j.jbc.2021.101077 PMC8424594

[CIT0010] Tsai JM, Nowak RP, Ebert BL et al Targeted protein degradation: from mechanisms to clinic. Nat Rev Mol Cell Biol 2024;25:740–757. 38684868 10.1038/s41580-024-00729-9

[CIT0011] Tsukahara M, Suemori H, Noguchi S et al Novel nucleolar protein, midnolin, is expressed in the mesencephalon during mouse development. Gene 2000;254:45–55. 10974535 10.1016/s0378-1119(00)00259-6

[CIT0012] Worden EJ, Dong KC, Martin A. An AAA motor-driven mechanical switch in Rpn11 controls deubiquitination at the 26s proteasome. Mol Cell 2017;67:799–811.e8. 28844860 10.1016/j.molcel.2017.07.023

[CIT0013] Xing Y, Clements WK, Kimelman D et al Crystal structure of a β-catenin/Axin complex suggests a mechanism for the β-catenin destruction complex. Genes Dev 2003;17:2753–2764. 14600025 10.1101/gad.1142603 PMC280624

[CIT0014] Zhao L, Zhao J, Zhong K et al Targeted protein degradation: mechanisms, strategies and application. Signal Trans Targeted Ther 2022;7:113. 10.1038/s41392-022-00966-4 PMC8977435 35379777

