# Supplementary material for: Structure-based engineering of the midnolin-proteasome pathway for targeted protein degradation
Source: Protein Cell. 2025 Aug 20;17(1):83–8. doi: 10.1093/procel/pwaf069 (PMC12888917; doi:10.1093/procel/pwaf069)
Supplement: pwaf069_Supplementary_Materials_1 [file pwaf069_supplementary_materials_1.pdf]

SUPPLEMENTAL MATERIAL

Materials and methods

Expression and purification of human midnolin-IRF4-26S proteasome complex

Full-length open reading frames of human midnolin were subcloned into pCAGGS vectors containing a C-terminal Flag tag. Full-length open reading frames of human IRF4 were subcloned into pCAGGS vectors with an N-terminal HA tag and a C-terminal Flag tag. The sequences were analyzed by SnapGene 4.2.4.

HEK293S GnTI$^-$ cells (ATCC) were cultured in Freestyle 293 medium (Thermo Fisher Scientific) supplemented with 1% (v/v) fetal bovine serum (ABW, XiaMen, China, www.mogengel.com). The cells were cotransfected with plasmids encoding human midnolin with or without IRF4 using PEI. Upon culture at 37°C for approximately 66 hrs, cells were incubated with 20 ng/mL phorbol 12-myristate 13-acetate (PMA; MedChemExpress, HY-18739) and 10 mM MG132 (Selleckchem, S2619) for 6 hrs. The cells were then harvested and lysed in lysis buffer containing 50 mM Tris-HCL (pH 8.0), 100 mM NaCl, 10% (v/v) glycerol, 5 mM ATP, 5 mM $MgCl_2$, 0.5% CHAPS, 1 mM DTT, 0.25 mM EDTA, and 1 mM PMSF supplemented with cOmplete™ Protease Inhibitor Cocktail (Roche) at 4°C for 30 min. The lysate was clarified by centrifugation at 26,000 g for 30 min at 4°C. The supernatant was incubated with Flag beads (GenScript) for 2 hrs, and proteins were eluted with buffer containing 20 mM Tris-HCL (pH 8.0), 100 mM NaCl, 10% (v/v) glycerol, 1 mM ATP, 1 mM $MgCl_2$, 0.25 mM EDTA, 1 mM DTT, and 0.2 mg/mL 3×Flag peptides. Eluted proteins were further

purified by an anion exchange Q column (GE Healthcare) equilibrated with buffer containing 20 mM HEPES (pH 8.0), 100-500 mM NaCl, 10% (v/v) glycerol, 1 mM $MgCl_2$, and 1mM DTT. The fractions containing midnolin-IRF4-26S or midnolin-26S proteasome were concentrated to 0.6 mg/mL, divided into aliquots, and flash-frozen in liquid nitrogen.

Purification of endogenous human 26S proteasome

A modified human embryonic kidney cell line HEK293T, expressing RPN11 bearing a Hexahistidine, TEV cleavage site, biotin and hexahistidine (HTBH) tag at C terminus, was used for proteasome purification (Wang et al., 2007). HEK293T cells were cultured on 150 × 25 mm dishes (BIOFIL) in Dulbecco's modified Eagle's medium (DMEM) containing 10% (v/v) FBS, 1% (w/v) pen/strep (GE Healthcare), at 37°C under 5% CO_2 and 85% humidity in a CO_2 incubator (Memmert). The cells were harvested by scraping, centrifugation at 500 g for 5min, and resuspension in lysis buffer containing 50 mM Tris-HCl (pH 8.0), 50 mM NaCl, 10% (v/v) glycerol, 5 mM ATP, 5 mM $MgCl_2$, 0.5% (v/v) NP-40, 1 mM DTT, and cOmplete™ Protease Inhibitor Cocktail (Roche). The lysate was homogenized by 100 strokes in a Dounce homogenizer and incubated for 15 min on ice, and centrifuged at 26,000 g at 4°C for 10 min. The supernatant was incubated with 2 mL High Capacity NeutrAvidin Agarose resin (Thermo) at 4°C overnight. After extensive washing with lysis buffer, the resin was cleaved by Tobacco Etch Virus (TEV) protease at 30°C for 3 hrs in buffer containing 50 mM Tris-HCl (pH 7.5), 1 mM EDTA, 1 mM ATP, and 1 mM DTT. The proteasome complex in the flow-through was concentrated and further purified by gel filtration on a Superose 6 increase

10/300 GL column with buffer 20 mM HEPES (pH 7.5), 50 mM NaCl, 50 mM KCl, 10% (v/v) glycerol, 2 mM ATP, 2 mM $MgCl_2$, 2 mM DTT. Finally, purified 26S proteasome was divided into aliquots and flash-frozen in liquid nitrogen. This protocol is based on methodology described in a previous study (Huang et al., 2016).

Expression and purification of human RPN1 and midnolin constructs

Gene fragments encoding full-length human RPN1 protein, human midnolin UBL (28-105), Catch (106-337), and C-Helix (375-413) were subcloned into pGEX-4T-1 vectors containing an N-terminal TEV protease site for removal of GST tags. Briefly, *E. coli* BL21 (DE3) transformed with an expression plasmid was cultured in Luria broth (LB) at 37°C to an OD_{600} of ~ 0.8, and overexpression of recombinant proteins was induced by adding isopropyl β-D-thiogalactoside (IPTG) to a final concentration of 0.2 mM at 18°C for 16–18 hrs. Harvested bacteria were resuspended in lysis buffer (50 mM Tris-HCl (pH 7.5), 500 mM NaCl, 10% glycerol, 2 mM DTT) and homogenized via sonication on ice. Lysates were cleared by centrifugation at 26,000 g for 1 hr at 4°C, and the supernatants were incubated with Glutathione Sepharose 4B resin (GE Healthcare) for 2 hrs at 4°C. The mixtures were then loaded onto an empty column (to collect the resin), and washed with lysis buffer. GST-tagged proteins were eluted with lysis buffer containing 20 mM reduced glutathione. For obtaining proteins without GST tags, the proteins were eluted with lysis buffer after cleavage of GST tags with TEV protease at 4°C overnight. Proteins were further purified by size exclusion chromatography using a Superdex 200 Increase 10/300 GL column (GE Healthcare) equilibrated with buffer containing 20 mM Tris-HCl (pH 7.5), 150 mM NaCl, 10%

glycerol, 2 mM DTT.

Cryo-EM sample preparation and data collection

To prepare Cryo-EM samples, the midnolin-26S or midnolin-IRF4-26S proteasome samples were first diluted to 0.3 mg/mL in dilution buffer containing 20 mM HEPES (pH 7.5), 50 mM NaCl, 50 mM KCl, 2 mM $MgCl_2$, 2 mM DTT, 0.01% (v/v) NP40. An aliquot of 4 μL diluted protein sample was applied onto a glow-discharged gold grid with single-layer graphene (R1.2/1.3, 300 Mesh, BGI). The grid was blotted with a blot force of -1 for 2 s after a 25 s waiting period at 8°C and 100% humidity, and then plunge-frozen into liquid ethane with a Vitrobot (Thermo Fisher Scientific). Cryo-EM images were collected on a 300 kV Titan Krios microscope (Thermo Fisher Scientific) equipped with a K3 detector (Gatan). Automated data acquisition was performed with SerialEM at a nominal magnification of 22,500 ×, which yielded a final pixel size of 1.06 Å, and with defocus ranging from –1.2 to –2.2 μm. The exposure time was set to 3 s, and the total accumulated dose was 60 e/Å^2.

Image processing

For midnolin-IRF4-26S proteasome complex and midnolin-26S proteasome complex, a total of 34,428 micrographs and 16,831 micrographs were collected respectively. Motion correction and the contrast transfer functions (CTF) were estimated by cryoSPARC patch motion correction and patch CTF estimation (Punjani et al., 2017). Particles were automatically picked on micrographs that were four-fold binned to a pixel size of 2.12 Å. A total of 2,522,203 raw particles were picked for midnolin-IRF4-26S proteasome complex and 4,090,907 raw particles were picked for midnolin-26S

proteasome complex. 1,606,388 particles and 2,407,753 particles were left for midnolin-IRF4-26S proteasome complex and midnolin-26S proteasome complex after 2D classification respectively, and used to perform ab initio reconstruction in five classes (Figs. S3). These classes were used as 3D volume templates for heterogeneous refinement with all selected particles. Then, particle subtraction and re-centering were performed and 3D classification was used to analyze the data. An RPN1-masked 3D classification was performed for MA and MD states. An RP-masked and a Catch domain-masked 3D classification was performed in order for the MB state. Poor 3D classes showing broken structures were removed. Final refinement of each state was performed with the pixel size of 1.06 Å. For the MB state, MB state with local Catch domain density impoved, and MD state, two types of masks were applied for the local refinement, one focusing on the complete RP and the other focusing on the CP, yielding two maps for each state, which were combined in Fourier space into one composite map. For MA state, the Fourier shell correlation (FSC) curves were calculated from two separately refined half maps in a gold-standard procedure, yielding the nominal resolution of 4.32 Å. The MB state yields the nominal resolution of 3.76 Å with local CP resolution at 3.43 Å and local RP resolution at 3.88 Å. The MB state with local Catch domain density impoved yields the nominal resolution of 4.52 Å, with local CP resolution at 4.17 Å and local RP resolution at 4.73 Å. The MD stateyields the nominal resolution at 3.31 Å, with local CP resolution at 3.18 Å; and local RP resolution at 3.71 Å.

Atomic model building and refinement

Atomic model building was based on the previously published cryo-EM structures of the human proteasome (Zhao et al., 2022, Dong et al., 2019). For the MA state, the initial model was derived from the E_{A1} model (PDB: 6MSB). For the MB state, the initial model was derived from the E_{B} model (PDB: 6MSE). For the MD state, the initial model was derived from the SD_{2} model (PDB: 8CVT). All subunits of the initial models were individually fitted as a rigid body into each of the reconstructed maps with UCSF Chimera (Pettersen et al., 2004), followed by further adjustment of the main chain traces using Coot (Emsley and Cowtan, 2004). Initial model of midnolin C-Helix and UBL domain were derived from the predicted structure of midnolin by AlphaFold2 (Jumper et al., 2021) and then merged with the initial proteasome model by fitting the model as a rigid body into the cryo-EM map.After manually rebuilding, atomic models were subjected to real-space refinement in Phenix (Adams et al., 2010). Partial rebuilding, model correction, and density-fitting improvement in Coot (Emsley and Cowtan, 2004) were then iterated after each round of atomic model refinement in Phenix (Adams et al., 2010) (Table S1).

Structural analysis and visualization

All structures were analyzed in Coot (Emsley and Cowtan, 2004), PyMOL(System), UCSF Chimera (Pettersen et al., 2004), and ChimeraX (Goddard et al., 2018). The interface areas were computed and analysed using the PISA server (Krissinel and Henrick, 2007) (https://www.ebi.ac.uk/pdbe/prot_int/pistart.html). The structure figures were plotted in PyMOL (System) or ChimeraX (Goddard et al., 2018).

Cell culture, transfection, immunoprecipitation

HEK293T cells were grown in DMEM (Gibco), 10% (v/v) FBS (Lonesera) at 37°C, 5% CO_2. Before transfection, cells were inoculated in 10 cm dishes and transiently transfected with 5 μg of indicated plasmids using Lipofectamine 3000 (Thermo Fisher Scientific) when reaching 70-80% confluency. Two days after transfection, the cells were treated with 20 ng/mL PMA, and 10 mM MG132 for 6 hrs. The cells were then rinsed once with ice-cold PBS and collected by scraping in 0.7 mL of lysis buffer containing 0.5% CHAPS, 40 mM HEPES (pH 7.5), 100 mM NaCl, 4 mM EDTA, 2 mM DTT, supplemented with cOmplete™ Protease Inhibitor Cocktail (Roche). Cell lysates were incubated with end-over-end rotation at 4°C for 30 min before clarification by centrifugation at 21,000 g, 4°C for 15 min. Flag beads were rinsed three times in lysis buffer and 20 μL of Flag beads were used for every harvested plate. A 20 μl aliquot of the cell lysate was collected as input, and the remaining supernatant was incubated with the beads for 2 hrs at 4°C. The beads were washed three times with the lysis buffer. Protein was then eluted by lysis buffer containing 0.2 mg/mL 3×Flag peptide. The cell lysates and immunoprecipitates were resuspended in SDS loading buffer containing 20% DTT, heating at 95°C for 10 min before analysis of protein content by immunoblotting.

GST pull-down assay

For GST pull-down assays, 5 μM of GST proteins, GST-midnolin C-Helix proteins and 10 μM of RPN1 proteins were mixed with 20 μL Glutathione Sepharose 4B resin (GE Healthcare) in 50 μL of pull-down buffer containing 20 mM Tris-HCl (pH 7.5), 150 mM NaCl, 10% glycerol and 10 mM DTT. The mixtures were incubated at 4 °C for 2 hrs. Then, the resin was washed three times with 200 μL of pull-down buffer. After

washing, the resin was eluted with pull-down buffer containing 20 mM reduced glutathione and analyzed by SDS-PAGE with Coomassie blue staining.

Western blotting

The protein samples in SDS loading buffer were analyzed by SDS-PAGE. Proteins were transferred to PVDF membranes (Millipore) using the Trans-Blot Turbo Transfer system (Bio-Rad). Membranes were blocked in 5% (w/v) milk (BD) in buffer containing 50 mM Tris-HCl (pH 7.4), 150 mM NaCl, 0.5% Tween20, and probed at room temperature (RT) for 1.5 hrs with primary antibody: rabbit polyclonal β-catenin antibody (Proteintech, 51067-2-AP, 1:10000 dilution), rabbit polyclonal histone-3 antibody (Proteintech, 17168-1-AP, 1:5000 dilution), mouse monoclonal GAPDH antibody (Proteintech, 60004-1-Ig, 1: 10000 dilution), rabbit polyclonal RPN1 antibody (Proteintech, 14748-1-AP, 1:2000 dilution), rabbit polyclonal Lamin B1 antibody (Proteintech, 12987-1-AP, 1:10000 dilution), HRP-conjugated Flag tag monoclonal antibody (Proteintech, HRP-66008, 1:10000 dilution), respectively, followed by incubation for 1 hrs at room temperature with HRP goat anti-Rabbit IgG (H + L) (Abclonal, AS014, 1:5000 dilution) or HRP goat anti-Mouse IgG (H + L) (Abclonal, AS003, 1:5000 dilution). Blots were developed with Clarity Western ECL Substrate (Bio-Rad) and exposed with Amersham Imager 680 Chemiluminescent Imaging System.

BLI assay

BLI assays were performed using the Octet RED96 system (Sartorius) to study the physical interactions between human RPN1 and human midnolin C-Helix. All

experiments were performed at RT, and the GST biosensors were pre-equilibrated in buffer containing 20 mM Tris-HCl (pH 7.5), 150 mM NaCl, 10% (v/v) glycerol, 10 mg/mL BSA, 0.02% Tween20, 10 mM DTT for at least 10 min. GST-tagged full-length hRPN1 was loaded onto GST biosensors (Sartorius). GST biosensors were then dipped into a solution containing midnolin C-Helix for binding measurements. The concentration gradients of midnolin C-Helix used in BLI assays were 1000 nM, 500 nM, 250 nM, 125 nM, 62.5 nM. The interference patterns from free GST-immobilized biosensors with the same concentration gradients were analyzed as controls. The binding affinities were determined using Octet Data Analysis 10.0 and final data analysis was done in GraphPad Prism 10.

Generating MIDN KO HEK293T cells using CRISPR/Cas9

To generate MIDN KO cells, HEK293T cells were initially transfected with the plasmid pX458 encoding CAS9-GFP and guide RNAs targeting genomic loci of the MIDN gene (Ran et al., 2013). Fluorescent cells were sorted by fluorescence-activated cell sorting (AriaIII, BD Biosciences), and individual clones were grown in 96-well plates. Deletions were validated by Sanger sequencing (Fig. S8I).

***In Vivo* Protein degradation assay**

For degradation *in cis*, different degron fragments were cloned into a pCAGGS vector, which possess N-terminal or C-terminal of eGFP, following a P2A site and mCherry using Gibson assembly technology. For degradation *in trans*, different degron fragments possessing N-terminal or C-terminal of eGFP nanobody were cloned into a pCAGGS vector, and the eGFP-P2A-mCherry plasmid was used as a protein

degradation reporter. MIDN KO HEK293T cells were transfected with indicated plasmids in an eGFP/mCherry protein degradation reporter vector. After 48 hrs, flow cytometry was used to quantify eGFP and mCherry fluorescence. The eGFP/mCherry fluorescence ratio was calculated using Cytexpert. For each experiment, at least three technical replicates and two experimental replicates were performed unless otherwise indicated.

Proteasome stimulating activity assay

The proteasome stimulating activity of wild-type midnolin or its variants or MidTAC toward human 26S proteasome was monitored by the cleavage of the fluorogenic peptide substrate Suc-LLVY-AMC (MedChemExpress). For the 26S proteasome stimulating activity assay, human 26S proteasomes (1 nM) were incubated with wt midnolin, midnolin-UBL, midnolin-C-Helix, midnolin-Catch, MidTAC-ICAT, MidTAC-BA, MidTAC-TB in buffer containing 50 mM Tris-HCl (pH 7.5), 100 mM KCl, 0.5 mM $MgCl_2$, 0.2 mM ATP, 2 mM DTT, 20 ng/μL BSA for 20 min at RT. 10 μM Suc-LLVY-AMC was added to the reaction mixture and incubated for 1 hr 30 min at RT. The fluorescent reaction product (AMC) was detected with a Spark multimode plate reader at 380 nm/460 nm (excitation/emission) for calculating the proteasome activity.

***In vitro* degradation assay**

0.6 μM of β-catenin and 1 μM of MidTAC-ICAT fusion proteins or its mutant MidTAC-ICAT (DQE) were incubated in the reaction buffer containing 50 mM Tris-HCl (pH 7.5), 25 mM NaCl, 25 mM KCl, 10% (v/v) glycerol, 10 mM $MgCl_2$, 5 mM ATP, 2 mM DTT, 0.5 mg/mL BSA for 30 min on ice. Then 5 nM purified human 26S proteasome

was added and incubated at 35°C. 50 μM MG132 was added to inactivate the 26S proteasome activity. 10 μL aliquots from each reaction were collected at indicated times. Collected samples were added with SDS-PAGE loading buffer, boiled immediately and stored at -20°C until used for SDS-PAGE analysis.

TOPFlash assay

One day before transfection, HEK293T cells were inoculated in 48-well plates at a concentration of 6×10^4 cells/well. 200 ng of the indicated plasmids, 60 ng of Wnt3a, 100 ng of M50 Super 8×TOPflash plasmid (#12456, Addgene) and 2 ng of CMV-Renilla plasmid were cotransfected into cells using Lipofectamine 3000 (Thermo Fisher Scientific) according to the manufacturer's instructions. The cells were harvested for luciferase reporter assays, which were performed according to the manufacturer's protocol (Dual Luciferase Assay kit, Promega). Since HEK293T cells were transfected with M50 Super 8×TOPflash plasmid, which contains a firefly luciferase cDNA driven by seven tandem repeats of the TCF binding site, Wnt activity was quantified by monitoring the activity of firefly luciferase. Renilla luciferase was used as an internal control. The luciferase activity was detected using Spark multimode microplate reader (Tecan). Values of unstimulated wt HEK293T cells were set to 10.

Isolation of cell subcellular fractions

Cytoplasmic and nuclear proteins were isolated using Nuclear and Cytoplasmic Protein Extraction Kit (Proteintech, PK10014) according to the manufacturer's instructions. All procedures were performed at 4°C. Briefly, after transfected 2 days, 1×10^6 HEK293T cells were collected, washed twice with ice-cold PBS, and pelleted by centrifugation at

500 g for 5 min. Cytoplasmic proteins were extracted by resuspending the cell pellet in 100 μL of Reagent A (containing protease inhibitors), followed by vortexing and incubation on ice for 10 min. Then centrifuge at 6,500 g for 5 min to collect the supernatant which contains cytoplasmic proteins. The cell pellet was sequentially resuspended in 100 μL of Wash Buffer A and B, with centrifugation following each wash to retain the cell pellet. Subsequently, 10 μL of Reagent B was added, and the mixture was vortexed and incubated on ice for 10 min. This step was repeated three times. The lysate was centrifuged at 16,000 g for 10 min to collect the supernatant which contains nuclear proteins. The subcellular fractions were used for Western blotting analysis.

AlphaFold multimer predictions

Amino acid sequences of midnolin and its various substrates (IRF4 (UniProtKB: Q15306), EGR1 (UniProtKB: P18146), c-FOS (UniProtKB: P01100), FOSB (UniProtKB: P53539), respectively) were input into AlphaFold 2 or AlphaFold 3 for monomer or multimer prediction with default reference databases specified as in (Jumper et al., 2021, Abramson et al., 2024).

Cell transduction

Generation of all RKO and HCT116 stable cells was performed using lentiviral particles. Briefly, viral supernatant was transduced with 5 μg/mL LV-assistant (Azneta) into RKO and HCT116 cells for 48 hrs, and 3 μg/mL puromycin used to select positive transduced cells.

Colony formation assay

200 cells were seeded to each well of 24-well plate and incubated for about two weeks. Fresh medium was changed every 3 days. At the last day, the plate was washed and stained by crystal violet. For the quantification, 250 μL of 40% acetic acid was added to each well to dissolve crystal violet and measurement at OD 590nm.

Table S1. Cryo-EM data collection, refinement, and validation statistics

States	MA	MB	MD
EMDB	64103	64133	63592
PDB ID	9UF8	9UG9	9M2W
**Data collection and processing**			
Voltage (kV)	300	300	300
Magnification	22,500×	22,500×	22,500×
Electron exposure ($e^-/Å^2$)	60	60	60
Defocus range (μm)	-1.2 to -2.2	-1.2 to -2.2	-1.2 to -2.2
Pixel size (Å)	1.06	1.06	1.06
Symmetry imposed	C1	C1	C1
Initial particle images (no.)	4,090,907	4,090,907	2,522,203
Final particle images (no.)	22,331	108,120	468,422
Map resolution (Å)	4.32	3.76	3.31
FSC threshold	0.143	0.143	0.143
**Refinement and validation**			
Initial models	6MSB	6MSE	8CVT
Model resolution (Å)	6.68	3.88	3.41
FSC Threshold	0.5	0.5	0.5
Model composition			
Non-hydrogen atoms	103,379	103,692	98,427
Protein residues	13,383	13,463	13,191
Ligands	6	6	5
R.m.s. deviations			
Bond lengths (Å)	0.006	0.006	0.006
Bond angles (°)	1.263	1.284	1.061
Validation			
MolProbity score	2.12	2.24	2.27
Clashscore	13.14	15.66	16.68
Rotamers outliers (%)	0.40	0.44	0.28
Ramachandran plot			
Favored (%)	91.95	90.37	90.09
Allowed (%)	7.66	9.16	9.45
Outliers (%)	0.39	0.47	0.47

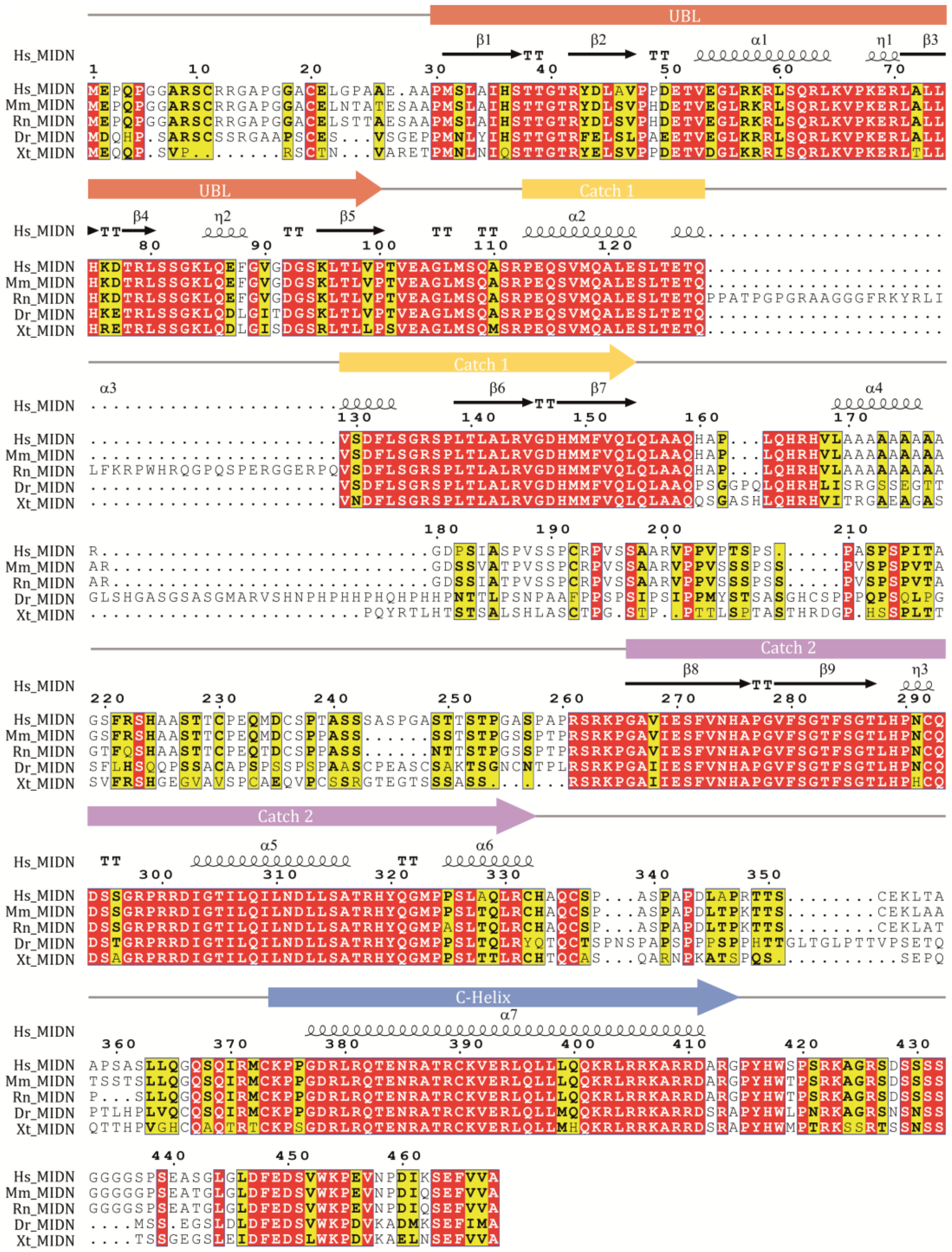

Figure S1 Sequence alignment of midnolin proteins from five different species using ESPript(Robert and Gouet, 2014). Secondary structural elements and domain composition of midnolin are indicated above the alignment. Abbreviations: Hs, *Homo sapiens*; Mm, *Mus musculus*; Rn, *Rattus norvegicus*; Dr, *Danio rerio*; Xt, *Xenopus tropicalis*.

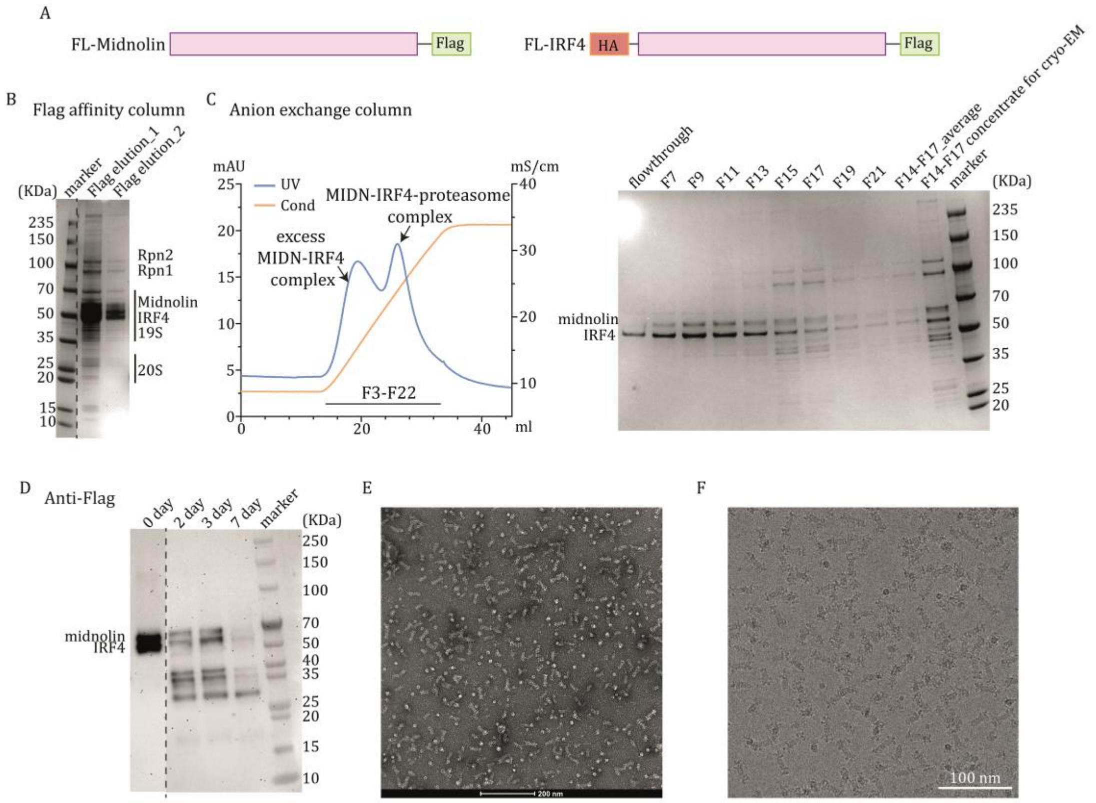

Figure S2 Protein purification and cryo-EM imaging of the midnolin-IRF4-proteasome complex. (**A**) Schematic of midnolin and IRF4 constructs used for transfection and protein expression. IRF4 is a native substrate recruited by midnolin. (**B, C**) Co-purification of the midnolin-IRF4-26S proteasome complex using Flag affinity chromatography and further purification by anion exchange chromatography, as shown by SDS-PAGE and the elution profile of the anion exchange column. The sample for cryo-EM preparation was labeled in SDS-PAGE (right). (**D**) The midnolin-IRF4 complex within the midnolin-IRF4-26S proteasome complex degraded over time, as probed by Flag antibody and shown by Western blotting. Almost all of the midnolin and IRF4 proteins in the cryo-EM sample preparation are intact, as shown in the first lane. (**E**) Typical negative staining micrograph of the midnolin-IRF4-26S proteasome complex. (**F**) Typical cryo-EM micrograph of the midnolin-IRF4-26S proteasome complex.

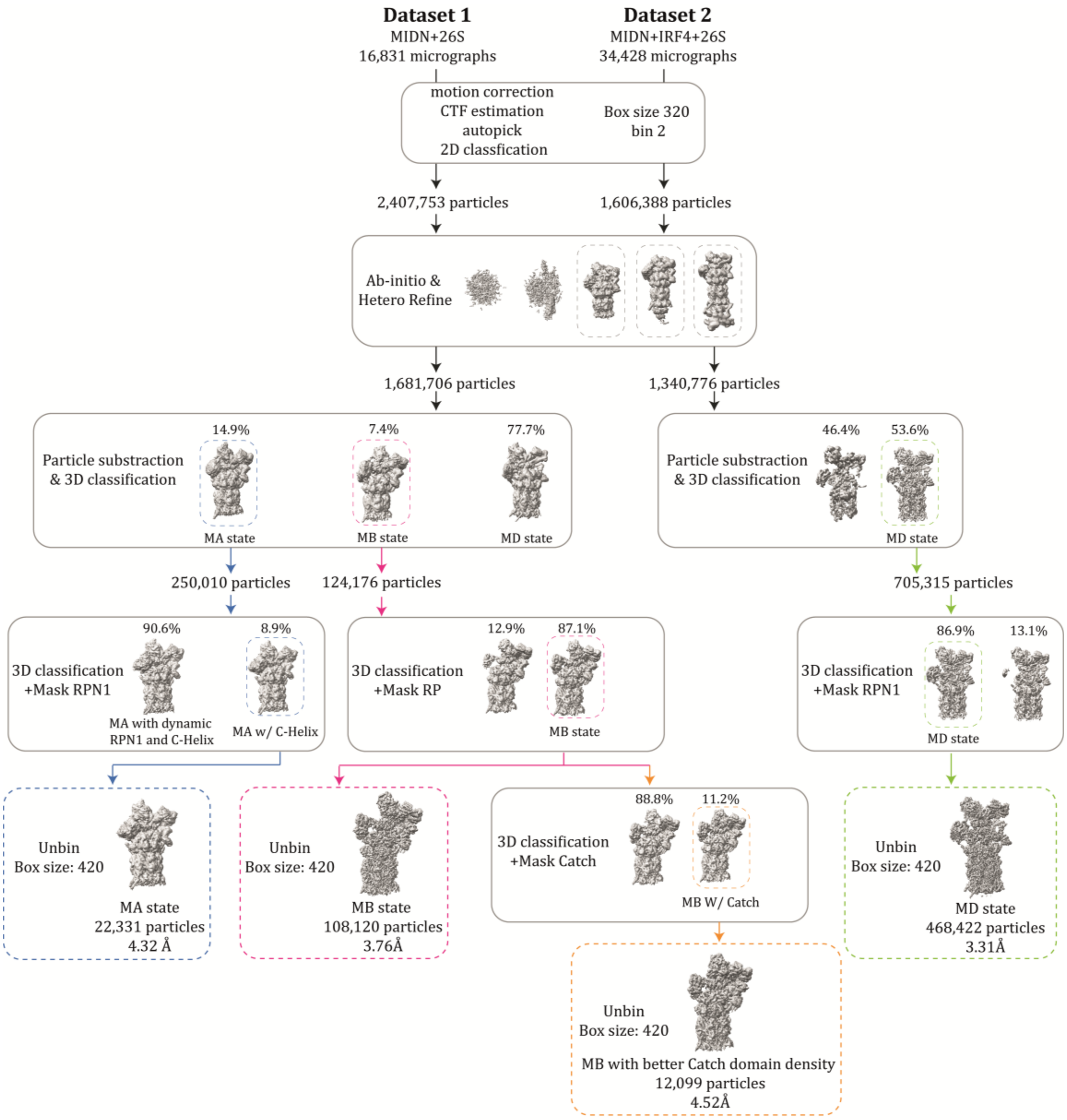

Figure S3 Cryo-EM data processing workflow for midnolin-proteasome and midnolin-IRF4-proteasome sample.

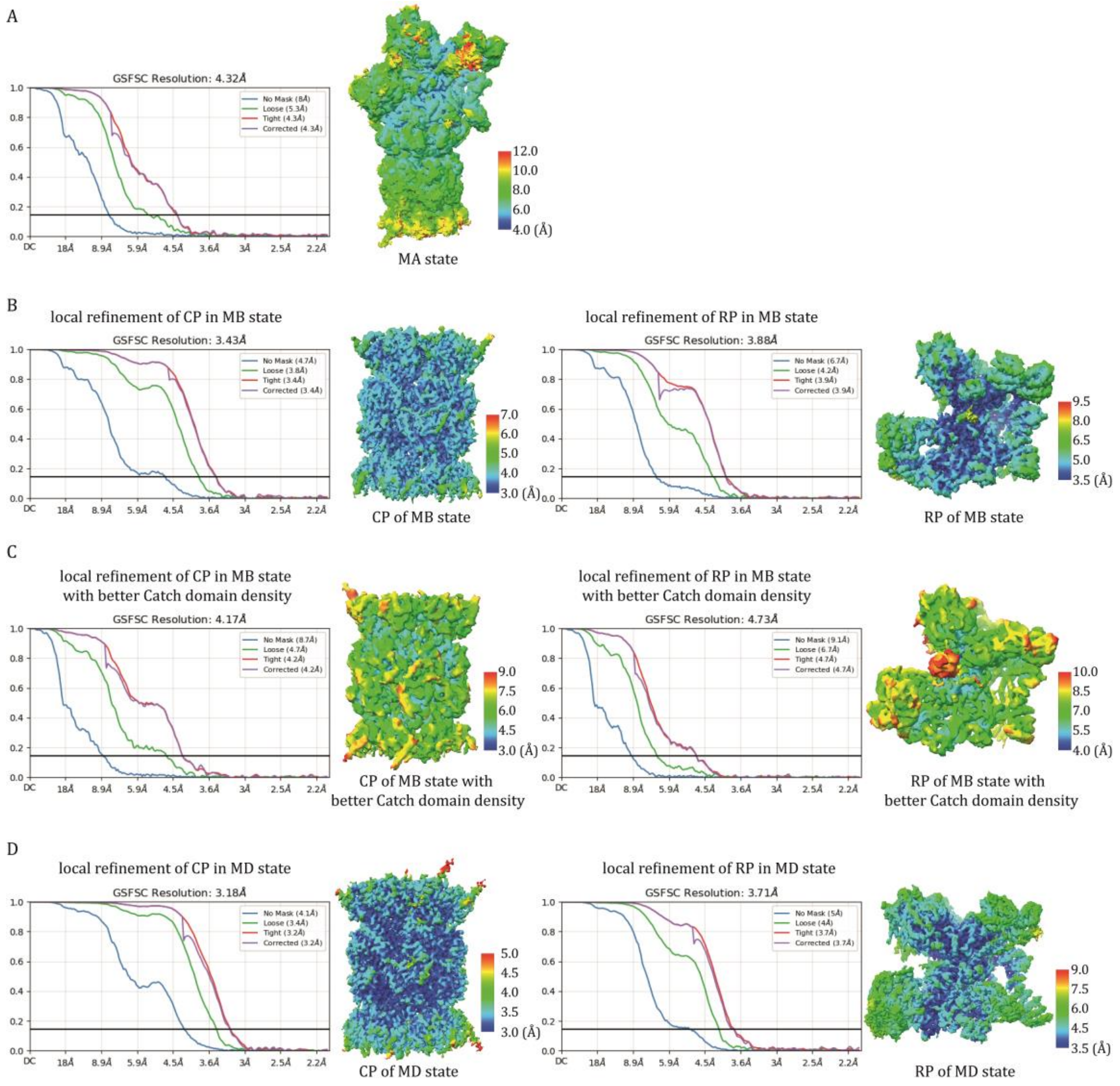

Figure S4 Cryo-EM reconstructions and resolution measurement. Gold-standard Fourier shell correlation (FSC) plots (left) and local resolution estimation (right) of the MA state (**A**), the CP (left) and RP (right) of the MB state (**B**), the CP (left) and RP (right) of the MB state with Catch domian density improved (**C**), and the CP (left) and RP (right) of the MD state (**D**) reconstructions calculated by local resolution estimation in CryoSPARC.

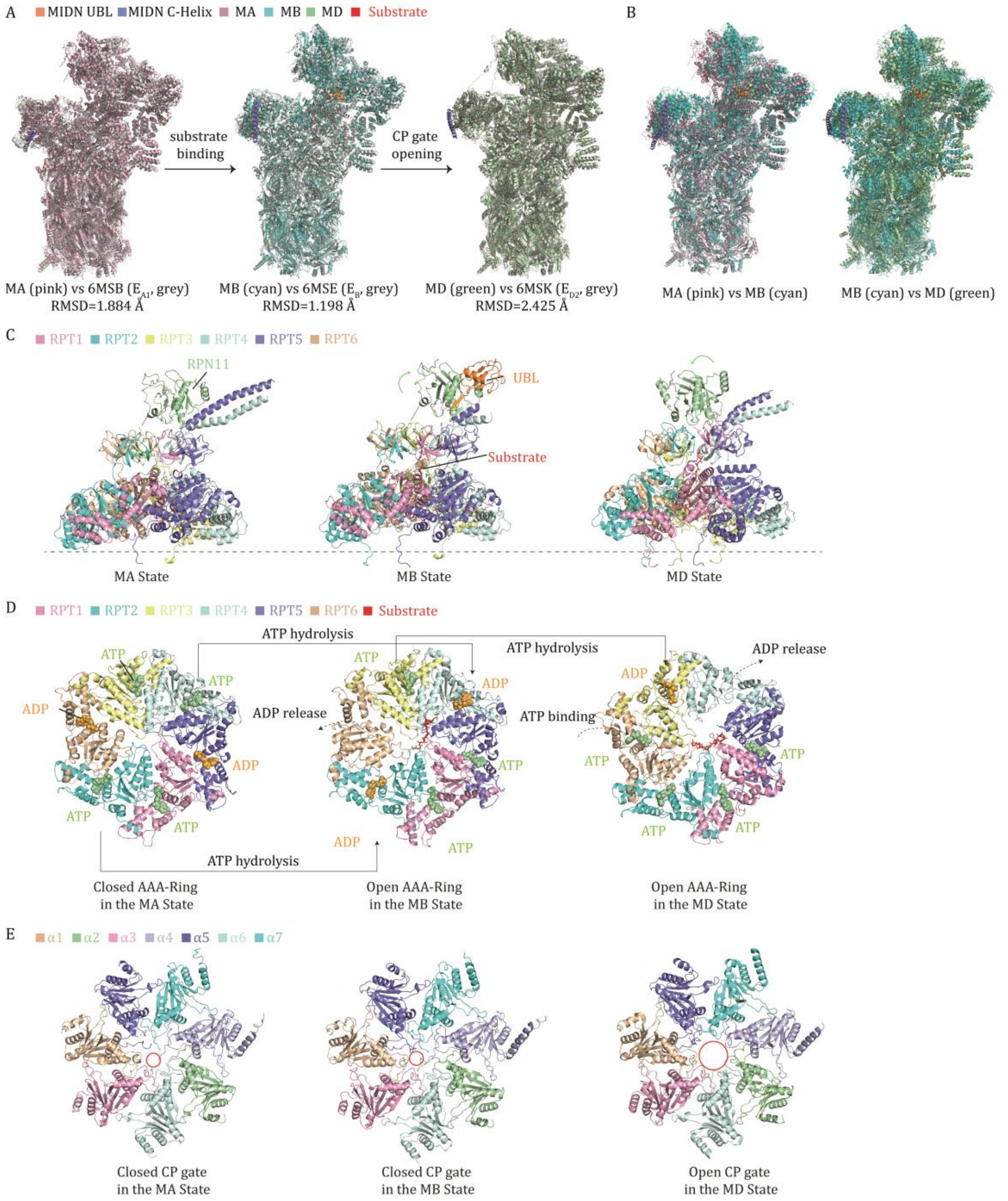

Figure S5 Structural comparisons of midnolin-26S proteasome complexes in the MA, MB and MD states. (**A**) Structure comparisons of MA and E_{A1} states (left), MB and E_B states (middle), MD and E_{D2} states (right). (**B**) Structure comparisons of MA and MB states (left), MB and MD states (right). (**C**) Side views of the ATPase subcomplex and RPN11 or MIDN-UBL in the MA, MB and MD states. The relative location of the CP is marked by the horizontal dashed line. (**D**) Top views of the ATPase motors of the MA, MB and MD states. Spheres representing ADP and ATP are shown in orange and

green, respectively. (**E**) Top views of the CP gates of the MA, MB and MD states.

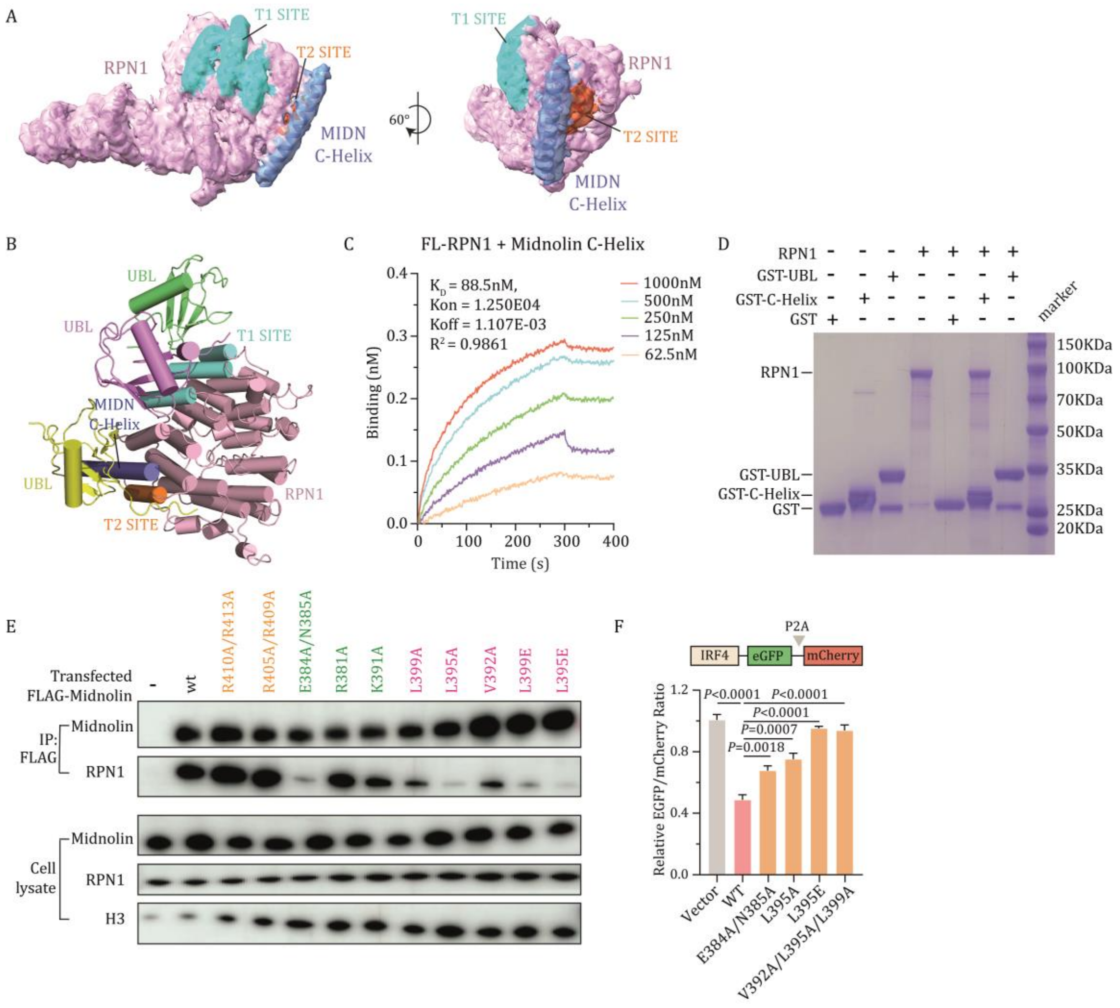

Figure S6 Midnolin C-Helix is necessary for its binding to the proteasome. (**A**) The density of MIDN C-Helix (blue) and RPN1 (pink) in the MD state. The T1 and T2 site of RPN1 are labelled as cyan and orange, respectively. It shows that MIDN C-Helix bound to a site very close to the RPN1 T2 site. The cryo-EM density is shown as a transparent surface overlaid with the cartoon representation of the atomic model. (**B**) Superposition of the structure of MIDN C-Helix (slate) and RPN1 complex with the structures of USP14 and RPN1 complex (PDB 7w37 (Zhang et al., 2022)), K48-linked diubiquitin and RPN1 complex (2N3W (Shi et al., 2016)). RPN1 T1 binding site was shown in cyan and RPN1 T2 binding site was shown in orange. (**B**) Binding affinity of MIDN C-Helix with full-length RPN1, as measured by BLI assays. (**C**) Midnolin C-Helix interacts with RPN1 *in vitro*, but midnolin UBL does not, as shown by GST pull-down assays. (**D**) The midnolin mutants bearing alanine substitutions of interface residues were precipitated with endogenous RPN1, as shown by co-IP assays. (**E**)

Midnolin requires residues mediating C-Helix and RPN1 interaction to promote degradation. The indicated wild-type full-length midnolin or its mutants were transfected into MIDN KO HEK293T cells with a IRF4-EGFP-P2A-mCherry plasmid. Protein stability was measured as EGFP/mCherry ratio. Empty vector was a control. Error bars indicate standard deviations; unpaired two-tailed t tests were used for statistical analyses.

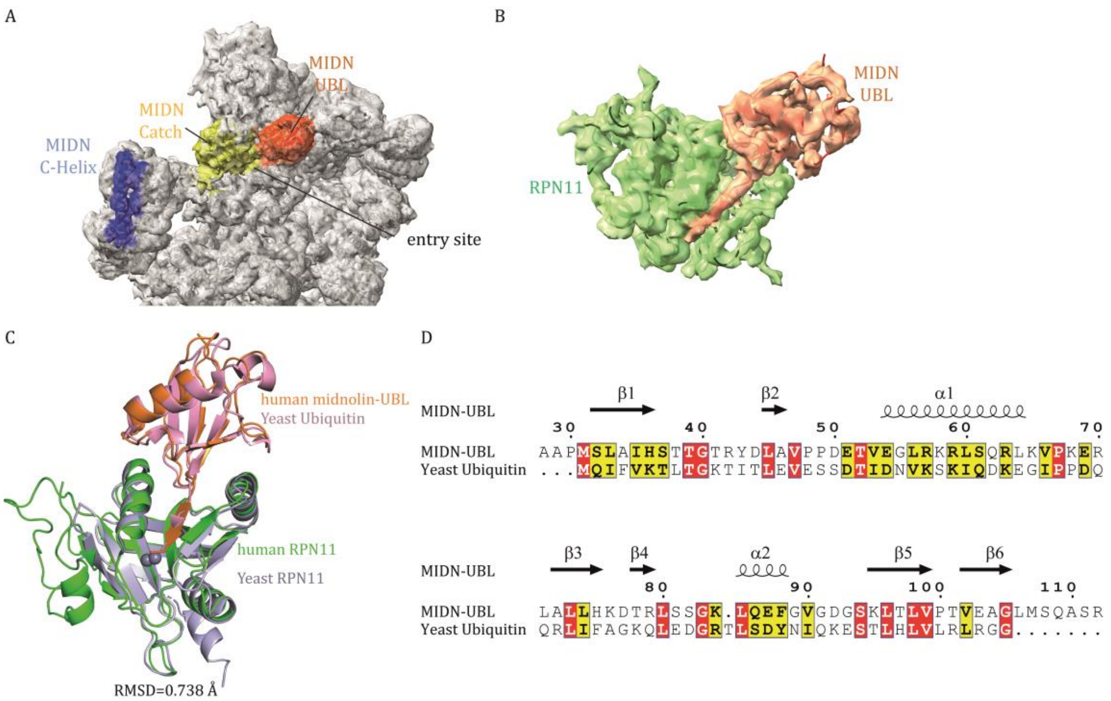

Figure S7 Midnolin UBL interacts with the 26S proteasome subunit RPN11. (**A**) The density of MIDN-UBL (orange), MIDN-Catch domain (yellow) and MIDN-C-Helix (Blue) in the MB state. It shows that Catch domain located above the AAA-ATPase entry site to facilitate substrate translocating and unfolding. The density for MIDN-Catch is not clear enough to allow precise model building. Thus, an AlphaFold3-predicted model for the Catch domain is roughly docked into the density. The cryo-EM density is shown as a transparent surface overlaid with the cartoon representation of the atomic model. (**B**) Zoom-in view of the density of MIDN-UBL (orange) and RPN11 (green) in the MB state. The cryo-EM density is shown as a transparent surface overlaid with the cartoon representation of the atomic model. (**C**) Superposition of midnolin UBL and RPN11 structure with yeast ubiquitin and RPN11 structure (Worden et al., 2017) (PDB: 5U4P). (**D**) Sequence alignment of human midnolin UBL domain and yeast ubiquitin using ESPript (Robert and Gouet, 2014). Secondary structural elements of midnolin UBL are indicated above the alignment.

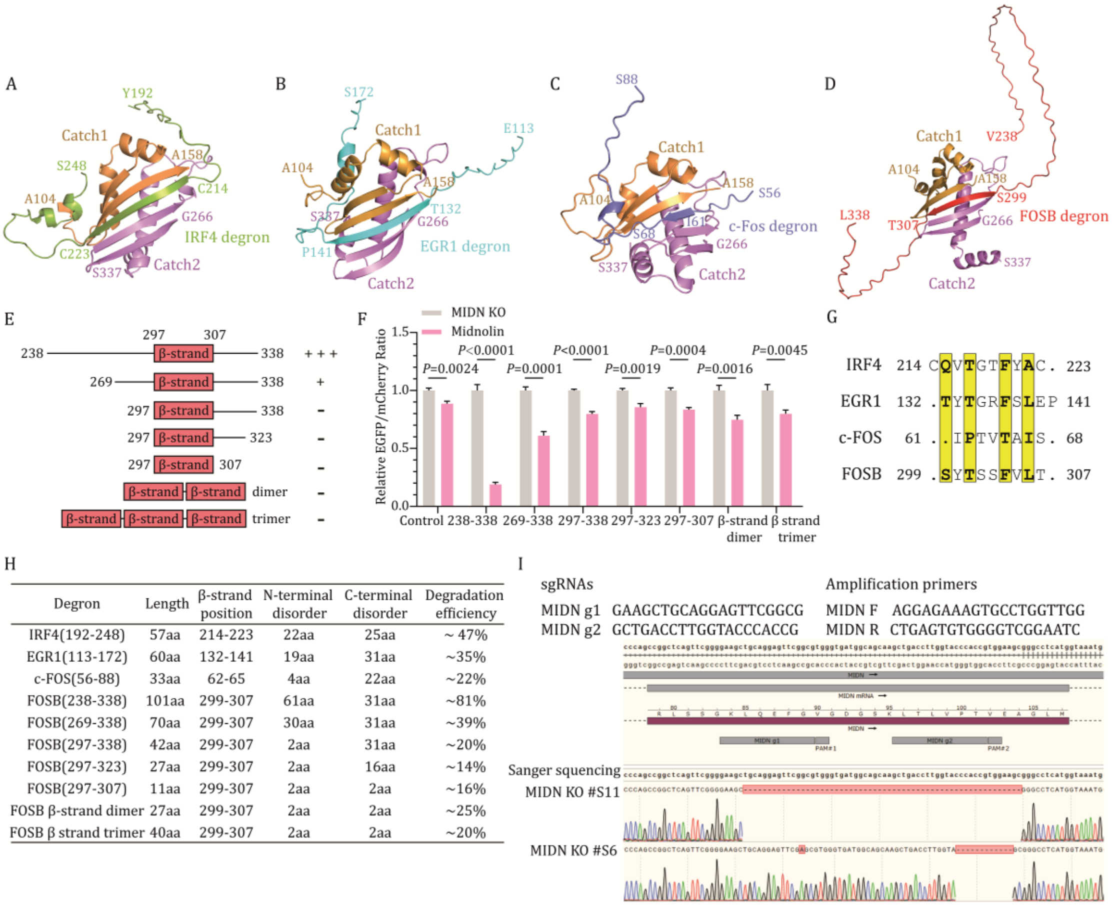

Degron	Length	β-strand position	N-terminal disorder	C-terminal disorder	Degradation efficiency
IRF4(192-248)	57aa	214-223	22aa	25aa	~47%
EGR1(113-172)	60aa	132-141	19aa	31aa	~35%
c-FOS(56-88)	33aa	62-65	4aa	22aa	~22%
FOSB(238-338)	101aa	299-307	61aa	31aa	~81%
FOSB(269-338)	70aa	299-307	30aa	31aa	~39%
FOSB(297-338)	42aa	299-307	2aa	31aa	~20%
FOSB(297-323)	27aa	299-307	2aa	16aa	~14%
FOSB(297-307)	11aa	299-307	2aa	2aa	~16%
FOSB β-strand dimer	27aa	299-307	2aa	2aa	~25%
FOSB β strand trimer	40aa	299-307	2aa	2aa	~20%

Figure S8 Midnolin degrons can function *in cis* and *in trans* for midnolin-dependent degradation. (**A-D**) AlphaFold-predicted structures of midnolin's Catch domain and IRF4 degron (**A**), EGR1 degron (**B**), c-FOS degron (**C**) or FOSB degron (**D**), respectively. (**E**) Schematic of FOSB degron truncations; + to +++, estimates of degradation efficiency; −, no degradation effect. (**F**) The degradation efficiency of different FOSB degron truncations. Similar assay as in (E, left). Error bars indicate standard deviations; unpaired two-tailed t tests were used for statistical analyses. (**G**) Sequence alignment of AlphaFold-predicted β-strands within four midnolin degrons. (**H**) Summary of degradation efficiency for different midnolin degrons fused to N-terminus of EGFP. (**I**) Generation of MIDN KO HEK293T cells using CRISPR/Cas9-based gene editing strategies. Disruption of MIDN gene was confirmed by sequencing for each knockout cell line.

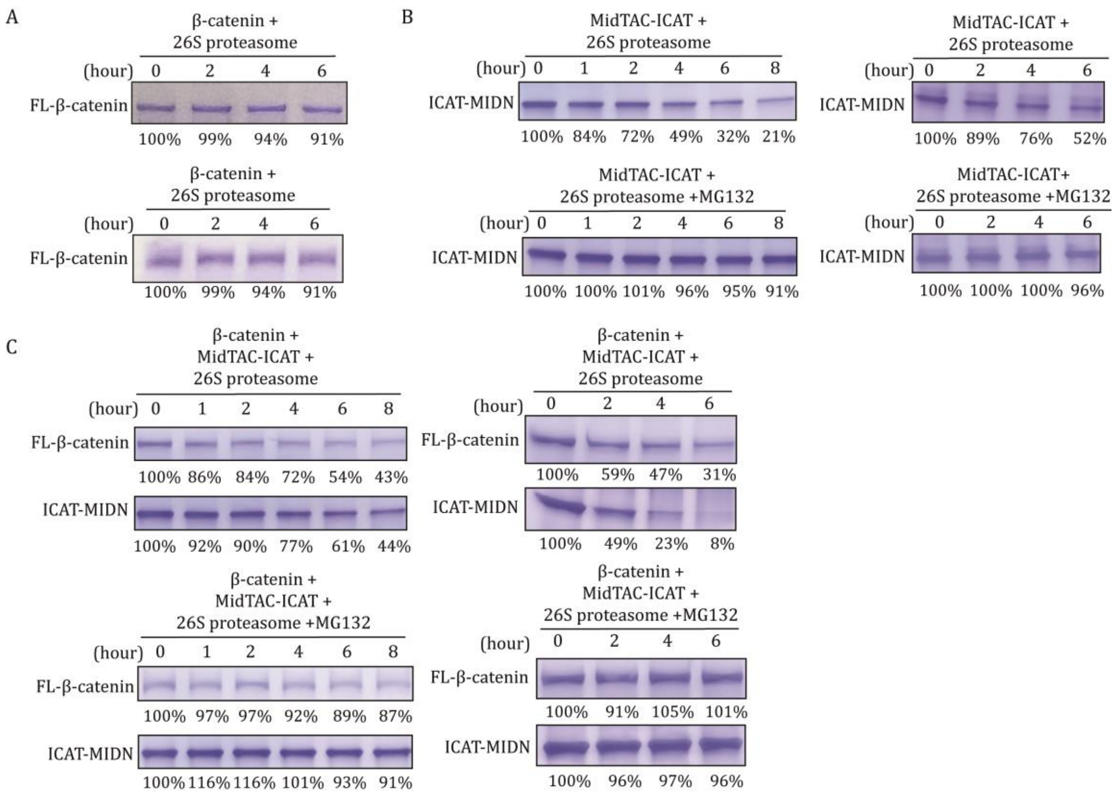

Figure S9 Engineered midnolin can promote targeted protein degradation *in vitro*. (**A**) *E. coli* purified β-catenin cannot be degraded by human 26S proteasome. Repeats 1 (top) and 2 (bottom). (**B**) *E. coli* purified engineered midnolin can be degraded by human 26S proteasome, and MG132 inhibits this degradation. Repeats 1 (left) and 2 (right). (**C**) *E.coli* purified engineered midnolin mediates degradation of *E.coli* purified β-catenin in the presence of purified human 26S proteasome, and MG132 inhibits this degradation. Repeats 1 (left) and 2 (right).

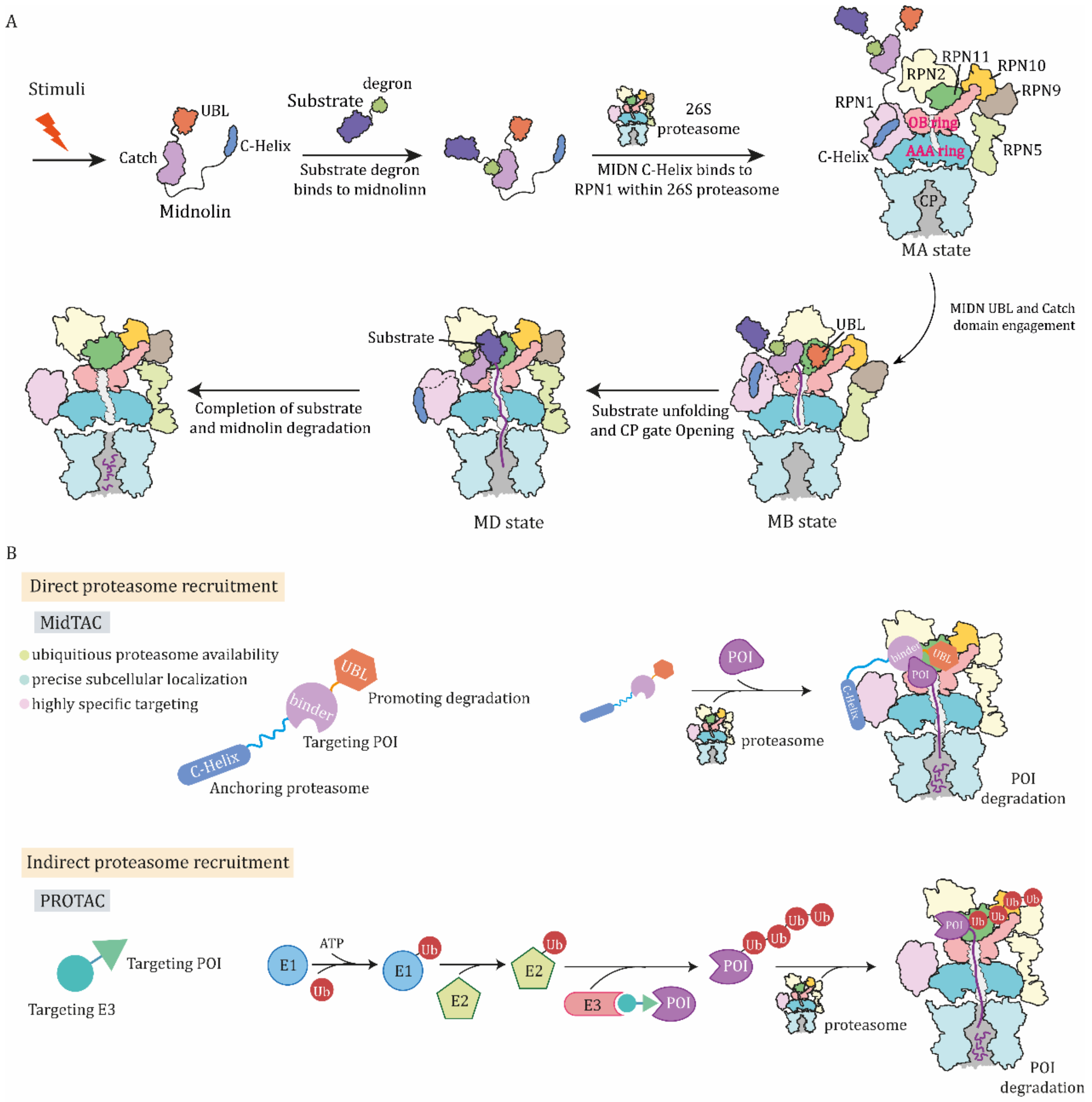

Figure S10 Mechanism-based engineering of the midnolin-proteasome pathway for targeted protein degradation. (**A**) The proposed model of the midnolin-proteasome pathway mediates substrate degradation in a ubiquitination-independent manner. When cells are stimulated, immediate-early genes (IEGs) and some transcription factors are rapidly upregulated. β-sheet-prone degrons in these substrates specifically bind to the subsequently expressed midnolin's Catch domain, forming the midnolin-substrate complex. Midnolin's C-helix directly interacts with the proteasomal subunit RPN1 to anchor the complex on proteasome (MA state). Then, midnolin's UBL domain interacts with RPN11, aligning the substrate-bound Catch domain above the entry site of the AAA-ATPase motor (MB state). This engagement widens the AAA-ATPase channel and facilitates substrate unfolding. Moreover, the translocation channel aligns with the

open CP gate to initiate processive substrate unfolding and translocation (MD state). Finally, the substrate is degraded. (**B**) Targeted protein degradation via direct or indirect proteasome recruitment. MidTAC, a heterobifunctional macromolecule, can simultaneously interact with proteasome subunits and the protein of interest (POI). It can induce POI degradation in one-step clearance via direct proteasome recruitment. PROTAC, a small heterobifunctional molecule, can simultaneously bind to the E3 ubiquitin ligase and POI. It can induce polyubiquitination formation on the POI and degrade the POI via the canonical ubiquitin-proteasome system (UPS).

References

ABRAMSON, J., ADLER, J., DUNGER, J., EVANS, R., GREEN, T., PRITZEL, A., RONNEBERGER, O., WILLMORE, L., BALLARD, A. J., BAMBRICK, J., BODENSTEIN, S. W., EVANS, D. A., HUNG, C.-C., O'NEILL, M., REIMAN, D., TUNYASUVUNAKOOL, K., WU, Z., ŽEMGULYTĖ, A., ARVANITI, E., BEATTIE, C., BERTOLLI, O., BRIDGLAND, A., CHEREPANOV, A., CONGREVE, M., COWEN-RIVERS, A. I., COWIE, A., FIGURNOV, M., FUCHS, F. B., GLADMAN, H., JAIN, R., KHAN, Y. A., LOW, C. M. R., PERLIN, K., POTAPENKO, A., SAVY, P., SINGH, S., STECULA, A., THILLAISUNDARAM, A., TONG, C., YAKNEEN, S., ZHONG, E. D., ZIELINSKI, M., ŽíDEK, A., BAPST, V., KOHLI, P., JADERBERG, M., HASSABIS, D. & JUMPER, J. M. 2024. Accurate structure prediction of biomolecular interactions with AlphaFold 3. *Nature,* 630, 493-500.

ADAMS, P. D., AFONINE, P. V., BUNKóCZI, G., CHEN, V. B., DAVIS, I. W., ECHOLS, N., HEADD, J. J., HUNG, L. W., KAPRAL, G. J., GROSSE-KUNSTLEVE, R. W., MCCOY, A. J., MORIARTY, N. W., OEFFNER, R., READ, R. J., RICHARDSON, D. C., RICHARDSON, J. S., TERWILLIGER, T. C. & ZWART, P. H. 2010. PHENIX: a comprehensive Python-based system for macromolecular structure solution. *Acta Crystallogr D Biol Crystallogr,* 66, 213-21.

DONG, Y., ZHANG, S., WU, Z., LI, X., WANG, W. L., ZHU, Y., STOILOVA-MCPHIE, S., LU, Y., FINLEY, D. & MAO, Y. 2019. Cryo-EM structures and dynamics of substrate-engaged human 26S proteasome. *Nature,* 565, 49-55.

EMSLEY, P. & COWTAN, K. 2004. Coot: model-building tools for molecular graphics. *Acta Crystallogr D Biol Crystallogr,* 60, 2126-32.

GODDARD, T. D., HUANG, C. C., MENG, E. C., PETTERSEN, E. F., COUCH, G. S., MORRIS, J. H. & FERRIN, T. E. 2018. UCSF ChimeraX: Meeting modern challenges in visualization and analysis. *Protein Sci,* 27, 14-25.

HUANG, X., LUAN, B., WU, J. & SHI, Y. 2016. An atomic structure of the human 26S

proteasome. *Nat Struct Mol Biol,* 23**,** 778-85.

JUMPER, J., EVANS, R., PRITZEL, A., GREEN, T., FIGURNOV, M., RONNEBERGER, O., TUNYASUVUNAKOOL, K., BATES, R., ŽíDEK, A., POTAPENKO, A., BRIDGLAND, A., MEYER, C., KOHL, S. A. A., BALLARD, A. J., COWIE, A., ROMERA-PAREDES, B., NIKOLOV, S., JAIN, R., ADLER, J., BACK, T., PETERSEN, S., REIMAN, D., CLANCY, E., ZIELINSKI, M., STEINEGGER, M., PACHOLSKA, M., BERGHAMMER, T., BODENSTEIN, S., SILVER, D., VINYALS, O., SENIOR, A. W., KAVUKCUOGLU, K., KOHLI, P. & HASSABIS, D. 2021. Highly accurate protein structure prediction with AlphaFold. *Nature,* 596**,** 583-589.

KRISSINEL, E. & HENRICK, K. 2007. Inference of macromolecular assemblies from crystalline state. *J Mol Biol,* 372**,** 774-97.

PETTERSEN, E. F., GODDARD, T. D., HUANG, C. C., COUCH, G. S., GREENBLATT, D. M., MENG, E. C. & FERRIN, T. E. 2004. UCSF Chimera--a visualization system for exploratory research and analysis. *J Comput Chem,* 25**,** 1605-12.

PUNJANI, A., RUBINSTEIN, J. L., FLEET, D. J. & BRUBAKER, M. A. 2017. cryoSPARC: algorithms for rapid unsupervised cryo-EM structure determination. *Nat Methods,* 14**,** 290-296.

RAN, F. A., HSU, P. D., WRIGHT, J., AGARWALA, V., SCOTT, D. A. & ZHANG, F. 2013. Genome engineering using the CRISPR-Cas9 system. *Nat Protoc,* 8**,** 2281-2308.

ROBERT, X. & GOUET, P. 2014. Deciphering key features in protein structures with the new ENDscript server. *Nucleic Acids Res,* 42**,** W320-4.

SHI, Y., CHEN, X., ELSASSER, S., STOCKS, B. B., TIAN, G., LEE, B. H., SHI, Y., ZHANG, N., DE POOT, S. A., TUEBING, F., SUN, S., VANNOY, J., TARASOV, S. G., ENGEN, J. R., FINLEY, D. & WALTERS, K. J. 2016. Rpn1 provides adjacent receptor sites for substrate binding and deubiquitination by the proteasome. *Science,* 351.

SYSTEM, T. P. M. G. 1.2r3pre ed.: Schrödinger.

WANG, X., CHEN, C. F., BAKER, P. R., CHEN, P. L., KAISER, P. & HUANG, L. 2007. Mass spectrometric characterization of the affinity-purified human 26S proteasome complex. *Biochemistry,* 46**,** 3553-65.

WORDEN, E. J., DONG, K. C. & MARTIN, A. 2017. An AAA Motor-Driven Mechanical Switch in Rpn11 Controls Deubiquitination at the 26S Proteasome. *Mol Cell,* 67**,** 799-811.e8.

ZHANG, S., ZOU, S., YIN, D., ZHAO, L., FINLEY, D., WU, Z. & MAO, Y. 2022. USP14-regulated allostery of the human proteasome by time-resolved cryo-EM. *Nature,* 605**,** 567-574.

ZHAO, J., MAKHIJA, S., ZHOU, C., ZHANG, H., WANG, Y., MURALIDHARAN, M., HUANG, B. & CHENG, Y. 2022. Structural insights into the human PA28-20S proteasome enabled by efficient tagging and purification of endogenous proteins. *Proc Natl Acad Sci U S A,* 119**,** e2207200119.
